# State-of-the-Art Molecular Dynamics Simulation Studies of RNA-Dependent RNA Polymerase of SARS-CoV-2

**DOI:** 10.3390/ijms231810358

**Published:** 2022-09-08

**Authors:** Shoichi Tanimoto, Satoru G. Itoh, Hisashi Okumura

**Affiliations:** 1 Institute for Molecular Science, National Institutes of Natural Sciences, Okazaki 444-8787, Aichi, Japan; 2 Exploratory Research Center on Life and Living Systems, National Institutes of Natural Sciences, Okazaki 444-8787, Aichi, Japan; 3 Department of Structural Molecular Science, SOKENDAI (The Graduate University for Advanced Studies), Okazaki 444-8787, Aichi, Japan

**Keywords:** molecular dynamics simulation, RNA-dependent RNA polymerase, SARS-CoV-2, nucleotide analogs, RNA replication inhibition

## Abstract

Molecular dynamics (MD) simulations are powerful theoretical methods that can reveal biomolecular properties, such as structure, fluctuations, and ligand binding, at the level of atomic detail. In this review article, recent MD simulation studies on these biomolecular properties of the RNA-dependent RNA polymerase (RdRp), which is a multidomain protein, of severe acute respiratory syndrome coronavirus 2 (SARS-CoV-2) are presented. Although the tertiary structures of RdRps in SARS-CoV-2 and SARS-CoV are almost identical, the RNA synthesis activity of RdRp of SARS-CoV is higher than SARS-CoV-2. Recent MD simulations observed a difference in the dynamic properties of the two RdRps, which may cause activity differences. RdRp is also a drug target for Coronavirus disease 2019 (COVID-19). Nucleotide analogs, such as remdesivir and favipiravir, are considered to be taken up by RdRp and inhibit RNA replication. Recent MD simulations revealed the recognition mechanism of RdRp for these drug molecules and adenosine triphosphate (ATP). The ligand-recognition ability of RdRp decreases in the order of remdesivir, favipiravir, and ATP. As a typical recognition process, it was found that several lysine residues of RdRp transfer these ligand molecules to the binding site such as a “bucket brigade.” This finding will contribute to understanding the mechanism of the efficient ligand recognition by RdRp. In addition, various simulation studies on the complexes of SARS-CoV-2 RdRp with several nucleotide analogs are reviewed, and the molecular mechanisms by which these compounds inhibit the function of RdRp are discussed. The simulation studies presented in this review will provide useful insights into how nucleotide analogs are recognized by RdRp and inhibit the RNA replication.

## 1. Introduction

**Figure 1:**
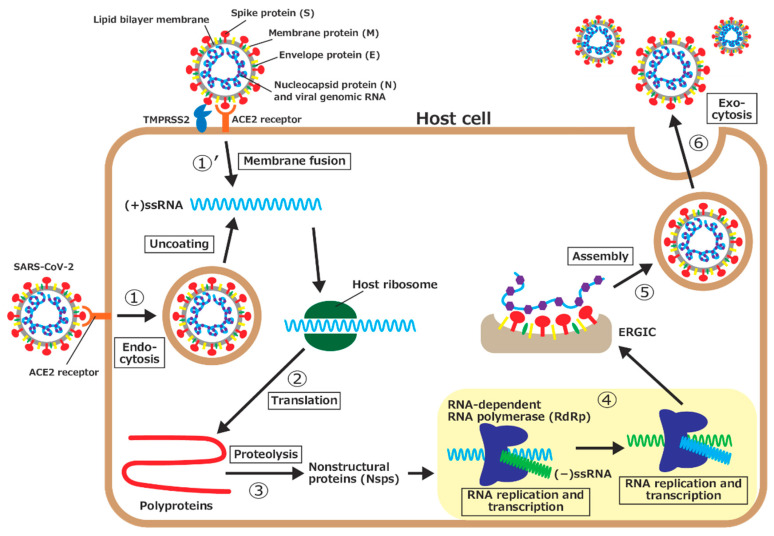
Schematic illustration of the SARS-CoV-2 life cycle in a host cell.

Coronavirus disease 2019 (COVID-19) is a disease caused by severe acute respiratory syndrome coronavirus 2 (SARS-CoV-2) [1]. This disease causes pathogenic symptoms such as fever, cough, and sore throat [2,3,4]. Seriously ill patients may develop a cytokine storm syndrome and pneumonia [2,5]. COVID-19 was first reported in Wuhan, China, in December 2019. It spread rapidly worldwide. In March 2020, the World Health Organization declared a pandemic of COVID-19 [6]. Similarly to other highly pathogenic viruses such as severe acute respiratory syndrome coronavirus (SARS-CoV) and Middle East respiratory syndrome coronavirus (MERS-CoV), SARS-CoV-2 is classified in the *Betacoronavirus* belonging to the family *Coronaviridae*. It has a large positive-sense single-stranded RNA ((+)ssRNA) genome consisting of about 30 kilobases, encoding more than 20 structural and nonstructural proteins (nsps) [7,8,9]. This virus invades a host cell and multiplies through the following process, as illustrated in Figure 1 [10]:

1. The spike protein on the surface of SARS-CoV-2 is bound to angiotensin-converting enzyme II (ACE2), a receptor on the host cell’s surface, and the virus enters the cell through endocytosis. The virus then uncoats, and the viral genomic RNA is released into the cytoplasm.
1’. Another way for the viral genomic RNA to enter the host cell is by membrane fusion. After the spike protein is bound to ACE2, part of the spike protein is cleaved by a type II transmembrane serine protease (TMPRSS2) on the host cell’s surface. The viral envelope is fused with the host cell’s membrane, and the viral genomic RNA is then released into the cytoplasm.
2. Because the genomic RNA of SARS-CoV-2 is (+)ssRNA, which functions as mRNAs, it is translated by ribosomes of the host cell. Two large proteins, called polyprotein 1a (pp1a) and polyprotein 1ab (pp1ab), are synthesized.
3. The polyproteins are then hydrolyzed, that is, proteolyzed, by virus-derived proteases, part of the polyprotein, to synthesize a series of nsps, which is necessary for the viral replication.
4. The RNA-dependent RNA polymerase (RdRp), one of the nsps, first synthesizes a negative-sense single-stranded RNA ((−)ssRNA) from (+)ssRNA. RdRp then uses (−)ssRNA as a template to synthesize the genomic RNAs for progeny viruses and several short subgenomic RNAs.
5. The synthesized nucleocapsid proteins are bound to the genomic RNA. In addition, various structural proteins are synthesized by translation of the subgenomic RNAs and inserted into the endoplasmic reticulum membrane. The genomic RNA and nucleocapsid protein complex is assembled with structural proteins (spike, membrane, and envelope proteins) at the endoplasmic reticulum–Golgi intermediate compartment (ERGIC) to form progeny viruses.
6. Progeny viruses are then released from the host cell through exocytosis.

**Figure 2:**
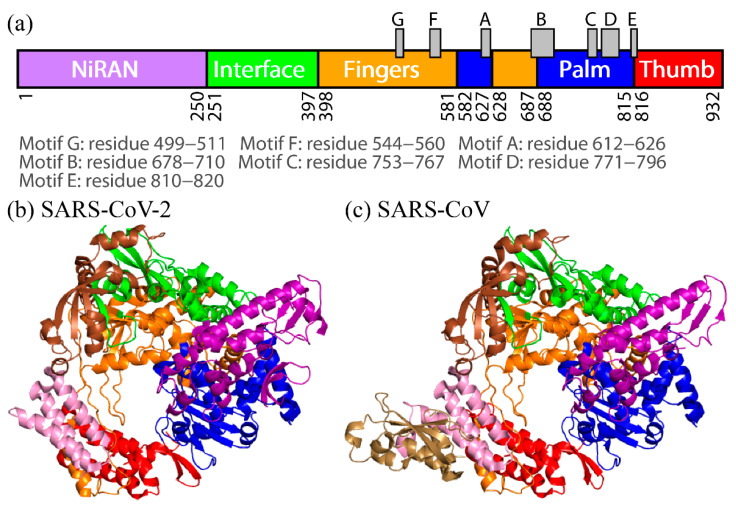
(**a**) Domains of SARS-CoV-2 nsp12 and motifs A–G. (**b**) The tertiary structure of SARS-CoV-2 RdRp and (**c**) that of SARS-CoV RdRp. These structures were determined by cryo-EM. NiRAN, interface, fingers, palm, and thumb domains are drawn in purple, green, orange, blue, and red, respectively. These colors correspond to those shown in (**a**). Nsp7 and two nsp8s (nsp8-1 and nsp8-2) cofactors are represented as pink, brown, and sand ribbons. Reproduced with permission from Ref. [20]. Copyright 2021 Elsevier.

Therapeutic agents and vaccines against COVID-19 have been developed thus far. One of the drug targets is RdRp [11,12]. The inhibition of RNA replications by therapeutic agents is expected to inhibit viral replications. RdRp of SARS-CoV-2 is a complex of nsp7, nsp8, and nsp12 [13,14,15,16]. The catalytic core of RdRp for the RNA replication is nsp12. However, nsp12 alone shows little activity [17,18]. Other subunits, such as nsp7 and nsp8, assist nsp12 as cofactors [17]. Nsp12 is a multidomain protein and has three main domains: nidovirus RdRp-associated nucleotidyltransferase (NiRAN) domain (residues 1–250), interface domain (residues 251–397), and conserved polymerase domain (residues 398–932) [19]. The polymerase domain consists of three subdomains: fingers, palm, and thumb. In the polymerase domain, seven motifs A–G, form the binding site of RdRp. The amino acids that constitute the domains and motifs are shown in Figure 2a.

**Figure 3:**
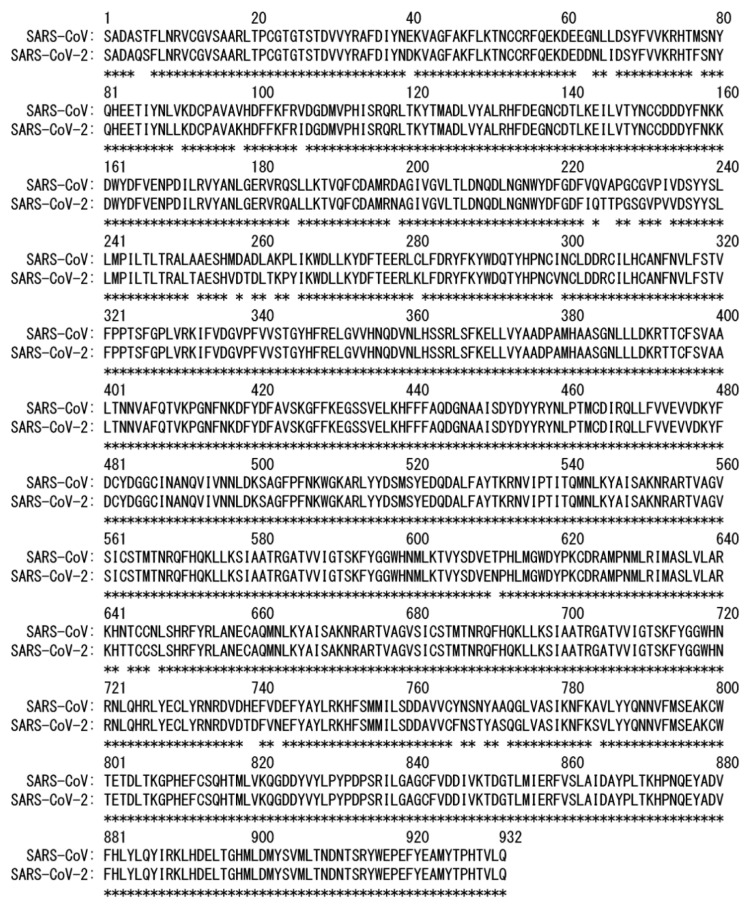
Amino-acid sequence of nsp12 of SARS-CoV-2 and that of SARS-CoV. The conserved amino-acid residues are represented by asterisks. Reproduced with permission from Ref. [20]. Copyright 2021 Elsevier.

The tertiary structure of RdRp consisting of nsp7, nsp8, and nsp12 was determined recently using cryogenic electron microscopy (cryo-EM), as shown in Figure 2b (Protein Data Bank (PDB) entry: 7bv2) [13]. In addition to this structure, other structures have also been reported. For example, the structure of RdRp bound to the RNA duplex [21], and the structures with RNA duplex and nsp13 helicase [22] were determined. The structure of RdRp in SARS-CoV-2 is almost identical to that of RdRp in SARS-CoV (Figure 2c, PDB entry: 6nur) [18]. Their nsp12s show more than 96% sequence identity [15,19]. The amino-acid sequences of these nsp12s are presented in Figure 3. Although the tertiary structures and amino-acid sequences of both nsp12s are almost identical, it has been reported that SARS-CoV-2 and SARS-CoV RdRps have different polymerase activities [23]. It has also been shown that replacing nsp12 of SARS-CoV-2 RdRp with nsp12 of SARS-CoV RdRp more than doubles polymerase activities.

**Figure 4:**
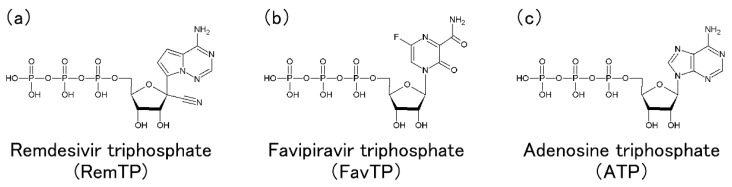
Chemical structures of (**a**) RemTP, (**b**) FavTP, and (**c**) ATP. Panels (**a**,**b**) were reproduced with permission from Ref. [24]. Copyright 2021 Biophysical Society.

In this review, we present computational molecular science studies on SARS-CoV-2 RdRp. This review is organized as follows. In Section 2 and Section 3, we describe the simulation studies that we have performed thus far on the dynamic properties and molecular recognition of RdRp. The difference in the activity of RdRp between SARS-CoV-2 and SARS-CoV is expected to be caused by the difference in their dynamic properties because the static properties, such as the tertiary structure, are almost the same. We introduce an all-atom molecular dynamics (MD) simulation study to investigate the difference in fluctuations of RdRp between SARS-CoV-2 and SARS-CoV in Section 2 [20]. Section 3 is devoted to an all-atom MD simulation study that elucidated the ligand-recognition process of RdRp in SARS-CoV-2 [24]. Nucleotide analogs such as remdesivir and favipiravir (brand name: Avigan) are drugs that target RdRps of the virus. Remdesivir was developed by Gilead Sciences (Foster City, CA, USA) originally for the Ebola virus disease [25]. Favipiravir was developed as an anti-influenza virus agent by Toyama Chemical (Tokyo, Japan) [26]. These drugs are thought to interfere with the RNA replications by RdRp, which normally recognizes nucleoside triphosphates (NTPs) such as adenosine triphosphate (ATP) for the RNA replication. Remdesivir is triphosphorylated (RemTP), and favipiravir is ribosylated and triphosphorylated (FavTP) in cells. Chemical structures of RemTP, FavTP, and ATP are illustrated in Figure 4. These forms are the active metabolite forms and are thought to inhibit the RNA replications by RdRp [13,27,28,29,30]. Note that remdesivir and favipiravir are not transformed to RemTP and FavTP directly but via several steps. Please refer to Ref. [31] for remdesivir transformations and Refs. [26,32] for favipiravir transformations in detail. We remark that RemTP should be referred to as GS-443902 [33], and FavTP should be referred to as favipiravir-ribofuranosyl-5′-triphosphate [34]. However, they are called RemTP and FavTP here for the sake of simplicity. In Section 4, we introduce various other simulation studies on the complexes of SARS-CoV-2 RdRp with several nucleotide analogs and discuss the molecular mechanisms by which these compounds inhibit the function of RdRp. In addition to the nucleotide analogs, MD simulations of RdRp with nonnucleoside antiviral compounds have been performed [35,36,37,38,39,40], but this review will focus on nucleotide analogs. Section 5 is devoted to the conclusions.

## 2. Difference in Dynamic Properties of SARS-CoV and SARS-CoV-2 RNA-Dependent RNA Polymerases

In this section, we introduce the MD simulation study of RdRp, which has been conducted to explore the reason for the difference in RdRp activities between SARS-CoV-2 and SARS-CoV. As mentioned in the Introduction, this difference is expected to be due to the difference in their dynamic properties because the static properties, such as the tertiary structure, of RdRps in SARS-CoV-2 and SARS-CoV are almost identical. It has been shown experimentally that there is a difference in the melting temperature between the two nsp12s [23]. However, the differences in the dynamic properties of the two RdRps had not been studied at the atomic level. Therefore, we performed all-atom MD simulations for RdRps of SARS-CoV-2 and SARS-CoV [20]. Note that the PDB structure of SARS-CoV-2 RdRp contains two Mg^2+^ ions that are essential for catalyzing RNA syntheses, while that of SARS-CoV RdRp does not contain Mg^2+^ ions. However, we also investigated the difference in dynamic properties with and without Mg^2+^ ions for SARS-CoV-2 RdRp, and we found that the structure and dynamics of RdRp did not change with the presence of the ions. We, thus, only present the results without Mg^2+^ ions here [20].

The cryo-EM structures of RdRp in SARS-CoV (PDB entry: 6nur) [18] and RdRp in SARS-CoV-2 (PDB entry: 7bv2) [13] were used as the initial structures. Na^+^ ions were added to neutralize the electric charge of the system. Water molecules were also included explicitly. The Amber parm14SB force field [41] was applied to proteins and ions. The TIP3P rigid body model [42] was used for water. The symplectic [43] quaternion scheme [44,45] was employed for water molecules. The particle mesh Ewald (PME) method [46,47] was used to calculate the electrostatic potential. MD simulations were performed in the isothermal–isobaric ensemble at 310 K and 0.1 MPa for 150 ns. The temperature and pressure were controlled using the Nosé–Hoover thermostat [48,49,50] and the Andersen barostat [51], respectively. To perform MD simulations, the Generalized-Ensemble Molecular Biophysics (GEMB) program was used. This program was developed by one of the authors (H. O.) and has been used for several biomolecules [52,53,54,55]. For other details, please refer to Ref. [20].

**Figure 5:**
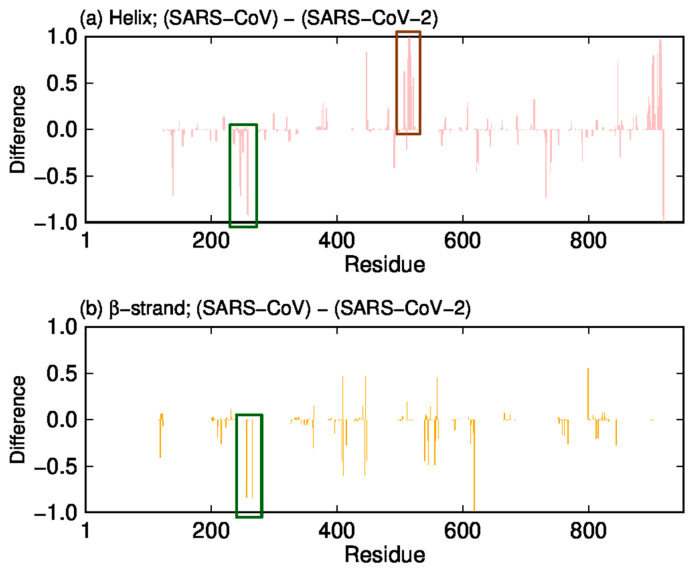
The difference between SARS-CoV nsp12 and SARS-CoV-2 nsp12 in formation probabilities of (**a**) helix and (**b**) β-strand structures. The green and brown rectangles indicate consecutive residues with large differences. Reproduced with permission from Ref. [20]. Copyright 2021 Elsevier.

We first calculated the formation probability of the secondary structure of RdRps using the Define Secondary Structure of Proteins (DSSP) criteria [56]. We then calculated the difference in the helix and β-strand formation probabilities between SARS-CoV and SARS-CoV-2. Figure 5a shows the difference in helix formation of nsp12, and Figure 5b shows the difference in β-strand formation. As highlighted by the green rectangles, the helix and β-strand structures are broken at residues near residue 260 in SARS-CoV nsp12. On the other hand, as highlighted by the brown rectangle, the residues near residue 515 of SARS-CoV nsp12 form more helix structures than SARS-CoV-2 nsp12.

**Figure 6:**
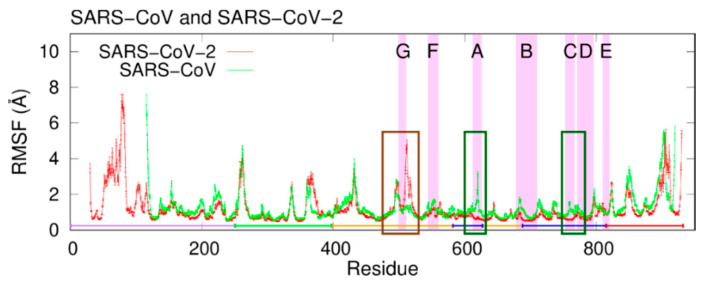
RMSFs of SARS-CoV nsp12 and SARS-CoV-2 nsp12. Purple, green, orange, blue, and red lines represent the NiRAN, interface, fingers, palm, and thumb domains, respectively. The violet-highlighted regions mean motifs A–G. The brown and green rectangles indicate residues that have large differences in RMSF. Reproduced with permission from Ref. [20]. Copyright 2021 Elsevier.

Next, the root-mean-square fluctuation (RMSF) was calculated to clarify the difference in the fluctuations of nsp12s. Figure 6 shows the RMSFs of SARS-CoV nsp12 and SARS-CoV-2 nsp12. The residues in the interface domain have large fluctuations in common to SARS-CoV-2 and SARS-CoV, except for the N- and C-terminal regions. However, the RMSF of SARS-CoV nsp12 differs from that of SARS-CoV-2 nsp12 near residues 515, 620, and 760, as indicated by the brown and green squares. The large fluctuations around residue 515 observed in SARS-CoV-2 nsp12 are suppressed in SARS-CoV nsp12. Parts of these residues constitute motif G. In SARS-CoV nsp12, residues near residues 620 and 760 have large fluctuations, and these residues exist in motifs A and C.

**Figure 7:**
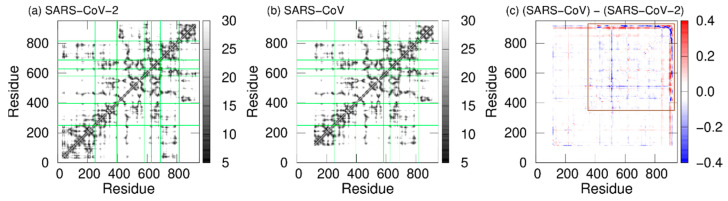
The average distances between C_α_ atoms of nsp12 for (**a**) SARS-CoV-2 and (**b**) SARS-CoV. The borders between the domains in nsp12 are indicated by the green lines. (**c**) The ratios of the difference between the average distances for SARS-CoV nsp12 and those for SARS-CoV-2 nsp12. The brown square shows residues that have large differences. Reproduced with permission from Ref. [20]. Copyright 2021 Elsevier.

Furthermore, to observe the tertiary-structure difference between SARS-CoV-2 nsp12 and SARS-CoV nsp12, the average distances between C_α_ atoms in nsp12s were calculated, as shown in Figure 7a,b. We can see that the two systems have the following in common: the NiRAN and palm domains are spatially close to each other, and the interface and fingers domains are close to each other. To clarify the difference in the average distances between SARS-CoV-2 nsp12 and SARS-CoV nsp12, we calculated the ratio of the difference as follows: $D_{ij}=\left(d_{ij}^{1}-d_{ij}^{2}\right)/d_{ij}^{1}$, where $d_{ij}^{1}$ is the average distance between the C_α_ atoms of residues *i* and *j* in SARS-CoV nsp12, and $d_{ij}^{2}$ is that in SARS-CoV-2 nsp12. The calculated ratios for the two systems are shown in Figure 7c. The difference between nsp12s of SARS-CoV-2 and SARS-CoV is observed in the region indicated by the brown square. Blue lines (or blue meshes) are observed in residues around 430, 520, 560, 620, 690, 760, and 800. These results mean that the distances between all motifs of nsp12 in SARS-CoV are shorter than those of nsp12 in SARS-CoV-2. In particular, the distance between motifs F and G for SARS-CoV is up to 63% shorter than that for SARS-CoV-2.

**Figure 8:**
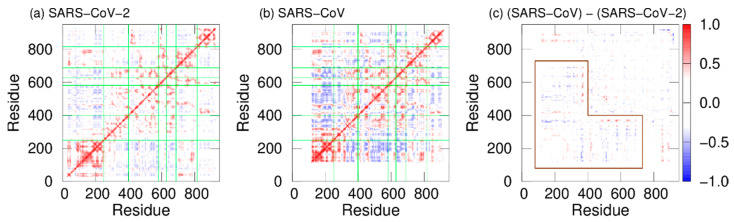
DCC of nsp12 for (**a**) SARS-CoV-2 and (**b**) SARS-CoV. The borders between the domains in nsp12 are indicated by the green lines. (**c**) Difference between DCC for SARS-CoV nsp12 and that for SARS-CoV-2 nsp12. The region surrounded by the brown lines means residues with large differences. Reproduced with permission from Ref. [20]. Copyright 2021 Elsevier.

In addition, dynamic cross-correlation (DCC) was calculated to investigate the correlation between domain motions. The DCC analysis is a useful tool to analyze domain motions of biomolecules [57]. DCC between residues *i* and *j* is defined by $DCC\left(i,j\right)=\langle \Delta q_{i}\cdot \Delta q_{j}\rangle /\sqrt{\langle {\left(\Delta q_{i}\right)}^{2}\rangle \langle {\left(\Delta q_{j}\right)}^{2}\rangle }$, where $\Delta q_{i}=q_{i}-\langle q_{i}\rangle$ and $q_{i}$ is the coordinate vector of the C_α_ atom of residue *i*. DCCs of SARS-CoV-2 nsp12 and SARS-CoV nsp12 are presented in Figure 8a,b. Here, red and blue indicate positive and negative correlations, respectively. The fact that there is a positive (negative) correlation between two residues indicates that the motions of these residues are in the same (opposite) direction. In both systems, positive correlations are found between most residues within the same domains. However, there are both positive and negative correlations in the interface domain of SARS-CoV nsp12. The boundary between these correlations is residue 330. Residues before and after residue 330 in the interface domain are positively correlated with the NiRAN domain and fingers domain, respectively. Figure 8c shows the difference in DCC between SARS-CoV-2 and SARS-CoV nsp12s. As shown by the region surrounded by the brown lines, the difference is larger in the NiRAN and interface domains. NiRAN and interface domains before residue 330 have a strong negative correlation with the fingers domain in SARS-CoV nsp12. That is, the regions before residue 330 move cooperatively with the fingers domain, moving closer and further away from each other.

The MD simulations described above show almost no differences in the tertiary structures of SARS-CoV nsp12 and SARS-CoV-2 nsp12 but some differences in their dynamic properties. The secondary structure near residue 260 of SARS-CoV nsp12 tends to be more broken than SARS-CoV-2 nsp12 (Figure 5). There are multiple substitutions in the residues near residue 260, as shown in Figure 3, and these differences in residues are thought to affect the stability of the secondary structure.

As shown in Figure 6, fluctuations of SARS-CoV nsp12 are suppressed around residue 515. This is because the residues near residue 515 form a helix structure, as shown in Figure 5a. On the contrary, the residues that constitute motifs A and C have larger fluctuations. In SARS-CoV-2 nsp12, fluctuations of the N-terminal residues before residue 100 are larger than the other residues. The structure of the N-terminal residues before residue 116 has not yet been determined in SARS-CoV nsp12, suggesting that the N-terminal residues of SARS-CoV nsp12 also have large fluctuations. As shown in Figure 7c, the distances between all motifs in SARS-CoV nsp12 are shorter compared to SARS-CoV-2 nsp12. This fact may also enhance the RdRp activity of SARS-CoV. Furthermore, in SARS-CoV nsp12, the NiRAN and fingers domains move cooperatively toward and away from each other; because the removal of the NiRAN domain reduces the RdRp activity [58], the NiRAN domain is important for the RdRp activities. The cooperative movement of the NiRAN domain with the core (fingers) domain of RdRp may enhance the activity of RdRp.

## 3. “Bucket Brigade” in RdRp Ligand Recognition

In this section, we review the ligand binding to SARS-CoV-2 RdRp. Because RdRp plays the most important role in the RNA replication, this protein is a promising drug target for COVID-19. Nucleotide analogs such as remdesivir and favipiravir are considered to compete with NTPs such as ATP to be taken up by RdRp and inhibit the RNA replication. However, the process by which the drugs and NTPs are incorporated into RdRp had been unclear. To clarify how RdRp takes up and recognizes the drugs and NTPs, we recently performed all-atom MD simulations of RdRp with RemTP, FavTP, or ATP [24]. The recognition ability of RdRp for each ligand was also clarified. Here, we explain this MD simulation study [24].

**Figure 9:**
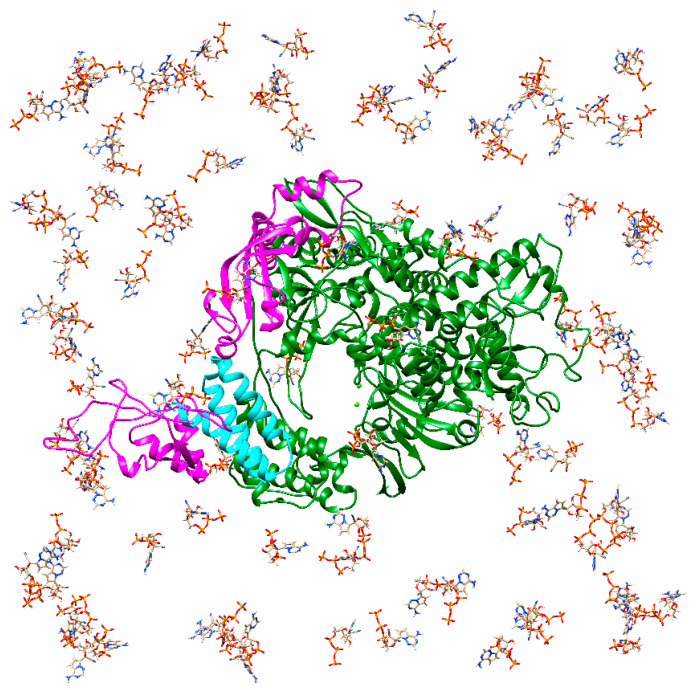
The initial structure of RdRp with 100 RemTPs. nsp12, two nsp8s, and nsp7 are represented as dark green, magenta, and cyan ribbons, respectively. The two small light-green spheres are Mg^2+^ ions. Reproduced with permission from Ref. [24]. Copyright 2021 Biophysical Society.

For the initial conformation of RdRp, the cryo-EM structure of RdRp of SARS-CoV-2 (PDB entry: 7bv2) was used [13]. Since one of the two nsp8s in this structure was missing, nsp8 corresponding to chain D of apo RdRp (PDB entry: 7bv1) [13] was added. One-hundred ligand molecules (either RemTP, FavTP, or ATP) were placed randomly around RdRp at a distance of at least 50 Å from one of the two Mg^2+^ ions at the binding site, as shown in Figure 9. Five initial conformations of each system were prepared by changing random seeds for the ligand arrangement. To neutralize the electric charge of the entire system, 415 Na^+^ ions were added. The Amber parm14SB force field [41] and TIP3P parameter [42] were used for proteins and water molecules, respectively. Under the periodic boundary conditions, long-range electrostatic interactions were calculated using the PME method [46,47]. The MD simulations were performed using the AMBER18 program [59]. The production run was performed for 110 ns from each initial condition. Fifty independent MD simulations were performed for each system with five different initial conformations and 10 different initial velocities. The MD simulations were performed at *T* = 310 K using the Langevin thermostat with a fixed volume.

As a result of the MD simulations, the ligands were taken up into the binding site of RdRp in all three systems of RemTP, FavTP, and ATP. In other words, the ligand recognition process by RdRp was observed. First, the ligand recognition probability was calculated, as listed in Table 1, to clarify the ligand dependence of the ligand recognition by RdRp. The ligand-recognition probability was calculated by dividing the number of MD simulations, in which a ligand recognition event occurred, by the total number of MD simulations. RemTP shows the highest probability, FavTP shows the second-highest probability, followed by ATP, although within statistical errors. These results are in qualitative agreement with previous experimental studies [13,60]. In addition, MD simulations of the RdRp-RemTP complex using the free energy perturbation method showed that RemTP is bound more strongly to RdRp than ATP [61], which is also consistent with the present results.

**Figure 10:**
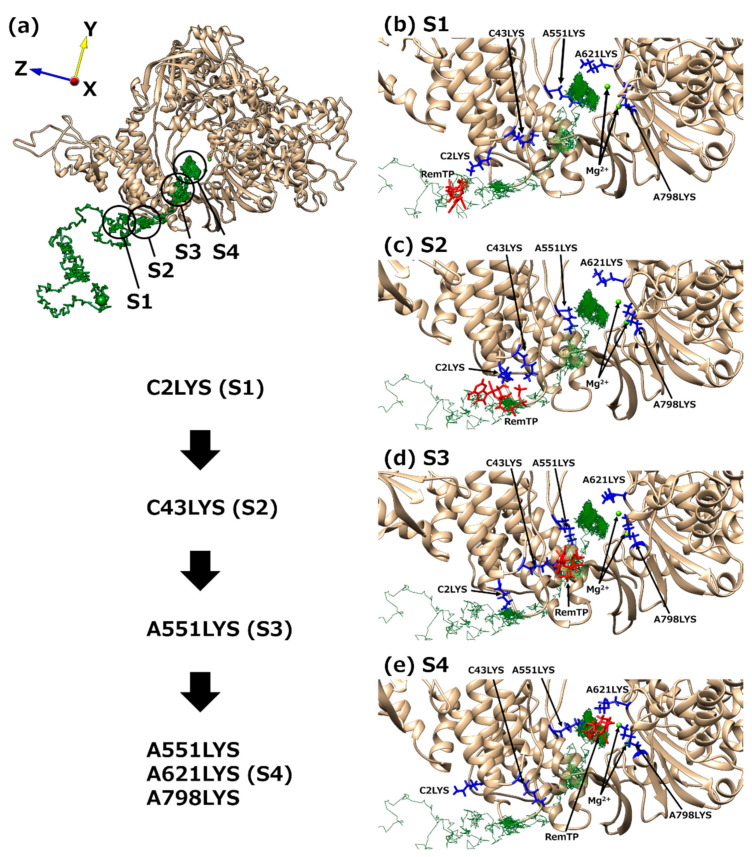
(**a**) “Bucket brigade” trajectory of RemTP recognized by RdRp. The black circles mean the positions at which RemTP has contact with RdRp residues. (**b**–**e**) Typical snapshot at each state (S1–S4). In (**b**–**e**), the lysine residues that contributed to the ligand recognition and RemTP are expressed as blue and red stick models, respectively. Reproduced with permission from Ref. [24]. Copyright 2021 Biophysical Society.

Next, to understand the mechanism of the ligand recognition by RdRp, the trajectories of the recognized ligands were examined. As a result, an interesting path was observed in which the lysine residues of RdRp carry ligands to the binding site like a “bucket brigade,” as shown in Figure 10. In this path, the phosphate groups of the ligands contacted LYS2 and LYS43 of nsp7 and LYS551, LYS621, and LYS798 of nsp12. Because nsp12 and nsp7 correspond to chain A and chain C, respectively, in the cryo-EM structure of the original PDB, the residues are expressed here as “chain label + residue number + residue name,” as written in Figure 10. These lysine residues have a positive charge, of which C2LYS, C43LYS, and A551LYS are in a line toward the binding site. In this process of ligand transportation, the phosphate groups of RemTP first interact with the side chain of C2LYS (state 1 (S1), Figure 10b). C2LYS passes RemTP to C43LYS, which is spatially close (state 2 (S2), Figure 10c). C43LYS then passes RemTP to A551LYS (state 3 (S3), Figure 10d). RemTP finally reaches the binding site (state 4 (S4), Figure 10e). The ligand also interacts electrically with A621LYS and A798LYS at the binding site. These residues are located near the binding site. A similar process was also observed in the FavTP and ATP systems. Another path has been found in which the ligand is transported to the binding site like a bucket brigade. For details, please refer to Ref. [24].

These positively charged basic residues have been reported to be favorable for the NTP recognition [13,15]. Furthermore, the lysine residues, A551LYS, A621LYS, A798LYS, C2LYS, and C43LYS, which contribute to the bucket-brigade ligand transportation, are highly conserved in RdRp of SARS-CoV [18]. Therefore, we think that for both SARS-CoV-2 and SARS-CoV RdRps, these linearly arranged lysine residues carry NTPs to the binding site, thereby enhancing the NTP recognition ability of RdRp.

**Figure 11:**
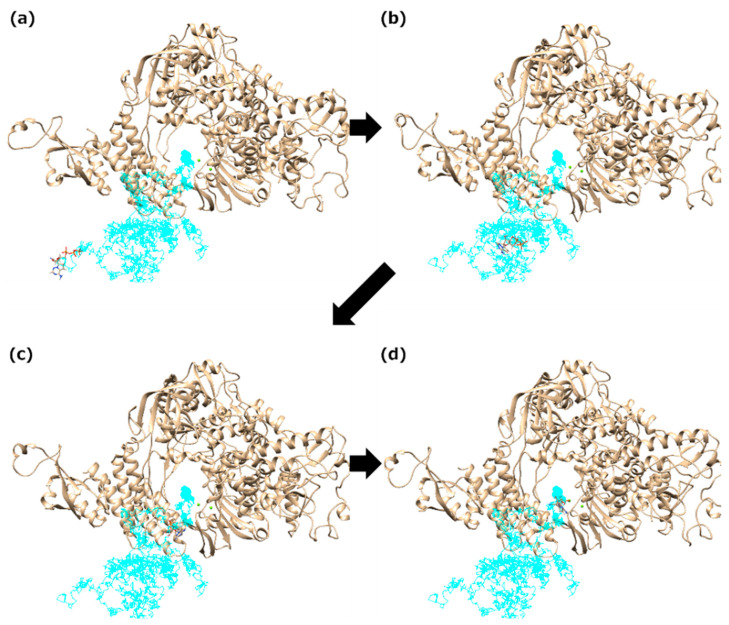
The direct trajectory of RemTP recognized by RdRp. (**a**–**d**) Typical snapshots in this trajectory. RemTP reaches the binding site without interactions with any residues of RdRp.

In addition to the bucket-brigade path of the ligand transport, we also found a path where the ligand reaches the binding site directly without interactions with any residues of RdRp (Figure 11). This path is the simplest process for the ligand recognition. The phosphate groups of the ligand are attracted to the two Mg^2+^ ions at the binding site by the electrostatic interaction. This path, as well as the bucket-brigade path, was observed in all ligand systems, suggesting that this is also one of the major ligand-recognition processes.

These paths are common to all ligands, but other ligand-specific paths were also found. In these paths, residues other than the positively charged residues near the binding site are involved in recognizing the ligands by RdRp. However, these paths may be minor because they are not common to all ligands.

## 4. Molecular Simulation Studies on Inhibition Mechanisms of Nucleotide Analogs against the SARS-CoV-2 RdRp Function

### 4.1. SARS-CoV-2 RdRp with Remdesivir in the Triphosphate Form

Experimental studies have shown that remdesivir is converted to the active triphosphate form (RemTP) by intracellular metabolism to be recognized by RdRp as a nucleotide analog [13,28,29]. It is important to understand the mechanism by which RemTP is bound to RdRp and inhibits the RNA replications. Zhang and Zhou performed MD simulations and free energy perturbation (FEP) calculations to examine the binding and inhibition mechanisms of RemTP against SARS-CoV-2 nsp12 [61]. They reported that RemTP was bound to nsp12 at about 100 times stronger in terms of the *K*_d_ value than ATP and suggested that RemTP replaces ATP and efficiently stops the RNA replications. Srivastava et al. performed a comparative simulation study of the apo-form of RdRp and the complex of RdRp with remdesivir, remdesivir monophosphate, or RemTP to understand the mechanism of the RdRp inhibition by RemTP [62]. They revealed that RemTP specifically interacted with the seven basic residues: LYS545, ARG553, ARG555, LYS621, ARG624, LYS798, and ARG836. They also identified three residues, SER549, ASP618, and PRO620, as the key contributors to RemTP binding. They suggested that the favorable binding of RemTP to these residues, especially LYS545, ARG553, and ARG555, interferes with the entry of new NTPs, which possibly perturbs the replication cycle. Romero et al. studied how RemTP is bound and inserted to the SARS-CoV-2 RdRp binding site with an RNA duplex using targeted MD simulations and umbrella sampling in comparison with ATP [63]. They reported that the insertion barrier for RemTP was ~1.5 kcal/mol and was lower than the barrier associated with the ATP insertion (2.6 kcal/mol). They also identified the residues involved in stabilizing the initial base stacking of RemTP with the template nucleotide and distinguishing between RemTP and ATP. Luo et al. performed MD simulations to elucidate the nascent-RNA-synthesis inhibition mechanism by remdesivir embedded in the template strand (T-Rem) [64]. It has been shown that T-Rem inhibits the synthesis of the nascent RNA strand [65]. They revealed that when T-Rem was at the binding site, the translocation of T-Rem was hampered by the hydrogen-bond formation between the 1′-cyano group of T-Rem and the backbone of GLY683 and the steric clash between the 1′-cyano group and the backbone of SER682. Olotu et al. reported the different binding behavior of RemTP and ATP to RdRp using MD simulations and molecular mechanics Poisson-Boltzmann surface area (MM-PBSA) calculations [66]. In addition to the higher affinity of RemTP for nsp12 than that of ATP, they found the novel mechanism that RemTP disintegrated RdRp, starting with the detachment of the nsp8-nsp7 heterodimer.

Zhang et al. examined how remdesivir integrated into the nascent RNA strand (N-Rem) inhibited RdRp from adding nucleotides to the strand [67]. They found that N-Rem could not impair the next nucleotide addition at the binding site of RdRp. However, they also revealed that N-Rem led to a delayed chain termination, where the translocation of the nascent RNA strand is terminated once three nucleotides were added after the RemTP incorporation. It was clarified that the forward translocation of the nascent RNA strand was impeded by the electrostatic repulsion between ASP865 and the 1′-cyano group of N-Rem as well as the steric clash between SER861 and the 1′-cyano group in a position where N-Rem reaches after three nucleotide incorporations. They also found that N-Rem at this site greatly weakened the hydrogen bonds of base pairs with its template uracil due to the electrostatic attraction between LYS593 and the 1′-cyano group. Their simulation study showed that the 1′-cyano group on the ribose was essential for remdesivir to inhibit the RNA replication by RdRp. Naseem-Khan et al. reported that the translocation of N-Rem was inhibited by the steric repulsion with SER861 using MD simulations [68]. Furthermore, the simulation study performed by Byléhn et al. showed base-pair hydrogen-bond interactions between template uracil and N-Rem weakened at three nucleotides upstream rather than at the 3′ terminal of the nascent RNA strand due to the strong electrostatic attraction between LYS593 and the 1′-cyano group of N-Rem [69]. Both Naseem-Khan et al. [68] and Byléhn et al. [69] also found that the overall structure of RdRp was destabilized by the uptake of remdesivir.

Using MD simulations and quantum mechanics/molecular mechanics (QM/MM) simulations for SARS-CoV-2 RdRp with an RNA duplex, Aranda et al. reported the detailed mechanisms of the binding and incorporation of natural nucleotides and RemTP [70]. They revealed that SARS-CoV-2 RdRp made use of a self-activated mechanism where the gamma-phosphate group of a pyrophosphate molecule deprotonated the hydroxylic 3′ terminal to generate the nucleophile that participated in the subsequent incorporation of a nucleotide. They also found that RemTP was preferentially bound to RdRp over ATP, while it was incorporated into the nascent RNA strand with an efficiency only slightly lower than ATP: the activation barrier of the phosphodiester bond formation was 16.2 kcal/mol for ATP and was 17.4 kcal/mol for RemTP. In addition, they reported that, unlike the results obtained through simulation studies by Zhang et al. [67] and Naseem-Khan et al. [68], no steric clash was detected between N-Rem and the residues of RdRp (especially SER861) when the nascent RNA strand was translocated along the exit channel. Instead, they found that N-Rem was trapped at a position where the three nucleotides were incorporated after RemTP. Therefore, they suggested that either non-covalent or transient-covalent bonds between the 1′-cyano group of N-Rem at this position and hydroxyl group of SER861 could act as a trap for the nascent RNA strand and stall the translocation of the duplex by stabilizing N-Rem.

### 4.2. SARS-CoV-2 RdRp with Other Nucleotide Analogs

Many simulation studies have been conducted on the inhibition mechanism of the RdRp function by nucleotide analogs other than remdesivir. Ribavirin 5′-triphosphate and FavTP were identified as promising nucleotide analogs using molecular docking, molecular mechanics Generalized Born surface area (MM-GBSA) calculations, and MD simulations [71]. Defant et al. synthesized several nucleoside-like compounds from a cellulose pyrolysis product and identified a nucleotide analog among those compounds that was more promising as RdRp inhibitors than remdesivir by molecular docking and MD simulations [72]. Sonousi et al. [73] and Elfiky et al. [74] identified several ATP derivatives and guanosine triphosphate (GTP) derivatives that had stronger affinities for RdRp than RemTP using molecular docking, MM-GBSA calculations, and MD simulations. Arba et al. performed molecular docking, MD simulations, and MM-PBSA calculations to study the binding mode and binding affinities of 3′ modified analogs of RemTP for nsp12 with an RNA duplex [75]. They reported that the RemTP analogs were bound to the nascent RNA strand in similar poses as that of RemTP with much higher affinities. They also found that the electrostatic contribution was the dominant factor in enhancing the binding affinity.

Yuan et al. investigated the incorporation efficiency and inhibitory effect of nucleotide analogs with various 2′ modifications against SARS-CoV-2 RdRp using MD simulations and FEP methods [76]. The nucleotide analogs included 2′-O-methyl uridine triphosphate (OMU-TP), sofosbuvir triphosphate (SFU-TP), 2′-C-methyl cytidine triphosphate (CMC-TP), Gemcitabine triphosphate (GMC-TP), and ara-uridine triphosphate (ARU-TP). Previous experimental studies reported that three of these, OMU-TP, SFU-TP, and CMC-TP, act as effective inhibitors, while GMC-TP and ARU-TP have no inhibitory effects [77,78,79]. Their simulation results showed that the five nucleotide analogs were effectively bound to RdRp with comparable binding affinities and were incorporated into the nascent RNA strand. They also revealed that OMU decreased the binding probability of the subsequent NTP and consequently caused partial chain terminations due to the steric hindrance by its 2′-O-methyl modification. In addition, it was found that the bulky 2′-methyl substitutions in SFU and CMC largely disrupted the binding site, leading to the immediate chain termination. In contrast, GMC and ARU, which have smaller 2′ substitutions such as the fluorine atoms and ara-hydroxyl group, showed marginal effects on the polymerization process upon the incorporation. Their simulation results were consistent with previous experimental results [77,78,79] and elucidated the detailed inhibition mechanisms of 2′ substituted nucleotide analogs against SARS-CoV-2 RdRp. Another simulation study also indicated the effectiveness of adding a bulky group at the 2′ position of the ribose ring to improve the inhibitory effects of nucleotide analogs [74].

Li et al. systematically investigated the inhibitory effects of ATP analogs possessing 2′ or 3′ ribose modifications against SARS-CoV-2 RdRp using MD simulations and FEP methods [80]. The analogs included clofarabine triphosphate (COP), didanosine triphosphate (DIP), fludarabine triphosphate (FLP), vidarabine triphosphate (VDP), 2′-amino-2′-deoxyadenosine triphosphate (BNP), 2′,3′-didehydro-2′,3′-dideoxyadenosine triphosphate (STP), and cordycepin triphosphate (CRP). Among them, COP, FLP, VDP, and BNP are the 2′ modified analogs, CRP is the 3′ modified analog, and DIP and STP are the 2′ and 3′ modified analogs. They found that clofarabine and fludarabine could not form stable binding at the binding site and only had a minor effect on the next nucleotide incorporation into the nascent strand. It was also clarified that vidarabine and 2′-amino-2′-deoxyadenosine could not efficiently inhibit the incorporation of the next substrate, although they could be incorporated into the nascent strand as the substrate. Didanosine, 2′,3′-didehydro-2′,3′-dideoxyadenosine, and cordycepin could also be incorporated into the nascent strand and had the capability to terminate the next nucleotide addition while STP was less competitive than the other two analogs. Therefore, they concluded that substituting the 3′-hydroxyl group with one hydrogen atom would inherently inhibit the next nucleotide addition when it appears at the 3′ terminal of the nascent strand. They proposed that cordycepin and didanosine were promising nucleotide analogs as immediate terminators.

### 4.3. Overview and Perspective of Molecular Simulations on SARS-CoV-2 RdRp with Nucleotide Analogs

NTPs and nucleotide analogs are integrated into the nascent RNA strand through a nucleotide-addition cycle consisting of initial binding to RdRp, uptake into the binding site, catalytic production of nucleotide monophosphate and pyrophosphate, the release of pyrophosphate, the formation of a covalent bond between nucleotide monophosphate and the 3′ end of the nascent RNA strand, the base pairing with the template strand, and the translocation of the RNA double strand. Nucleotide analogs inhibit the RNA replications by interfering with the addition of the next nucleotide (immediate chain termination) [77] or by interfering with the translocation of the nascent RNA strand after the incorporation of three nucleotides (delayed chain termination) [60]. It was shown that nucleotide analogs embedded in the template strand also inhibit the RNA replications (template-dependent inhibition) [65]. The simulation studies reviewed here elucidated the atomic level mechanisms of the delayed chain termination of remdesivir, immediate chain termination of 2′ and 3′ modified nucleotide analogs, and template-dependent inhibition of remdesivir. Furthermore, these simulation studies also suggested more promising nucleotide analogs for inhibiting the function of RdRp than remdesivir [72,73,74,75]. All simulation studies focused on the situation after NTPs or nucleotide analogs are incorporated into the binding site of RdRp. On the other hand, our simulation study described in Section 3 [24] focused on the process by which ligands far from RdRp were incorporated into the binding site and revealed the bucket-brigade transport mechanism of NTPs and nucleotide analogs by lysine residues of RdRp. Overall, the simulation studies described in this review help us in enhancing the understanding on how nucleotide analogs are recognized by RdRp and inhibit the RNA replication at the atomic level.

## 5. Conclusions

Molecular dynamics (MD) simulation is one of the powerful theoretical methods, which can reveal biomolecular properties such as structure, fluctuations, and ligand binding in atomic detail. This review article presented recent MD simulation studies on these biomolecular properties of RNA-dependent RNA polymerase (RdRp), which is a multidomain protein, of severe acute respiratory syndrome coronavirus 2 (SARS-CoV-2) and the inhibition mechanism of nucleotide analogs against RdRp. First, we reviewed the multiplication mechanism of SARS-CoV-2 and described the role of RdRp, which is composed of viral nonstructural proteins (nsps), nsp7, nsp8, and nsp12 [13,14,15,16,17,18,81]. RdRp normally recognizes nucleoside triphosphates (NTPs) such as adenosine triphosphate (ATP) to replicate RNA. The catalytic core of RdRp for the RNA replication is nsp12. SARS-CoV-2 nsp12 and SARS-CoV nsp12 show more than 96% sequence identity [15,19]. In addition, the tertiary structures of both nsp12s determined by cryogenic electron microscopy are almost the same. However, it has been reported that nsp12s of SARS-CoV-2 and SARS-CoV have different polymerase activities [23]. Replacing only nsp12 of SARS-CoV-2 with that of SARS-CoV, the activity of SARS-CoV-2 RdRp increases more than twice [23]. Since the static properties are almost the same between SARS-CoV-2 and SARS-CoV nsp12s, it is expected that a difference in their dynamic properties may cause an activity difference.

We introduced MD simulations of RdRps of SARS-CoV-2 and SARS-CoV, which were performed to investigate the difference in their dynamic properties. The MD simulations showed that the dynamic properties of SARS-CoV and SARS-CoV-2 nsp12s are different from each other: in SARS-CoV nsp12, the fluctuations near the residues that constitute motifs A and C are larger, and motifs A–G are closer to each other. Furthermore, the NiRAN and fingers domains move cooperatively in SARS-CoV nsp12. These differences may cause the difference in the activity of the two nsp12s. It has also been experimentally shown that the melting temperatures of these two nsp12s are different. Generalized-ensemble algorithms [82,83], such as replica exchange [84,85,86] and replica permutation methods [87,88,89,90], will be useful for future studies of the melting process.

We then presented an MD simulation study on SARS-CoV-2 RdRp and three ligands, GS-443902 (RemTP), favipiravir-ribofuranosyl-5′-triphosphate (FavTP), and ATP to clarify the recognition mechanism of these ligands. It was found that the recognition probability of RemTP is the highest, that of FavTP is the second highest, and that of ATP is the lowest. In addition, the “bucket-brigade” ligand-transport mechanism in the RdRp ligand recognition was discovered, in which these ligands are transported to the binding site by several lysine residues in a line. The lysine residues that interact with the phosphate groups of the ligands include LYS2 and LYS43 of nsp7, and LYS551, LYS621, and LYS798 of nsp12. On the other hand, there is another path in which the ligands directly reach the binding site without contacting any amino-acid residues of RdRp. The direct uptake of the ligands by RdRp means that RdRp can recognize NTPs without using the bucket-brigade mechanism by the lysine residues. RdRp has the lysine residues in a line toward the binding site as if it were extending its tentacles, thereby increasing the efficiency of the NTP recognition. These results are expected to contribute to understanding the efficient NTP recognition by RdRp and to developing drugs that inhibit the RdRp function. The residues identified in the simulations as contributing to the NTP recognition are well conserved in RdRp of SARS-CoV [18]. Therefore, we can extend these results to the NTP recognition mechanisms in other RNA viruses with similar RdRp to SARS-CoV-2.

This simulation study [24] revealed the process by which ligands far from RdRp were incorporated into the binding site. In addition, several MD simulations have been performed on SARS-CoV-2 RdRp after NTPs or nucleotide analogs were recognized, as reviewed in Section 4. These simulation studies have elucidated the mechanisms of the delayed chain termination of remdesivir, immediate chain termination of 2′ and 3′ modified nucleotide analogs, and the template-dependent inhibition of remdesivir.

In this manner, the MD simulations have provided valuable insights at the atomic level into the fundamental properties of apo RdRp and a sequence of the mechanisms from the uptake of NTPs or nucleotide analogs by RdRp to the inhibition of the RdRp function by nucleotide analogs, leading to the design of more promising drugs than remdesivir. We hope that MD simulations will be utilized more as effective tools for the detailed analysis of the functions of proteins involved in the SARS-CoV-2 replication and the development of therapeutically more effective drug compounds to contribute to the convergence of COVID-19.

## Figures and Tables

**Table 1:** The number of MD simulations in which RdRp recognized the ligands out of the total 50 MD simulations. Ligand recognition probability is also listed. Reproduced with permission from Ref. [24]. Copyright 2021 Biophysical Society.

Ligand	Ligand Recognition/Total	Ligand Recognition Probability
RemTP	12/50	0.24 ± 0.07
FavTP	9/50	0.18 ± 0.06
ATP	7/50	0.14 ± 0.06
